# Quantum–Chemical Multiligand Simultaneous Docking of Three-Membered Rings in the Active Site of Butyrylcholinesterase

**DOI:** 10.3390/cimb48040395

**Published:** 2026-04-13

**Authors:** Nika Jakobović, Petra Kalinovčić, Jakov Borovec, Ines Primožič, Tomica Hrenar

**Affiliations:** Department of Chemistry, Faculty of Science, University of Zagreb, Horvatovac 102a, 10000 Zagreb, Croatia

**Keywords:** multiligand docking, three-membered rings, BChE, fragment-based drug design

## Abstract

Alzheimer’s disease is a progressive neurodegenerative disorder marked by declining cognitive function. While early-stage treatment focuses on acetylcholinesterase (AChE) inhibition, butyrylcholinesterase (BChE) activity increases as the disease progresses, contributing to cholinergic deficits and neuroinflammation. This shift in enzyme dominance presents a compelling rationale for developing BChE-specific inhibitors as a potential therapeutic avenue. This study explores small, three-membered rings, scaffolds offering potential for interaction with the enzyme’s active site, as building blocks for novel BChE inhibitors. Employing a computational approach based on quantum–chemical multiligand simultaneous molecular docking, we virtually fitted these compounds into the BChE active site to predict binding affinity and key interactions. Our calculations extend beyond simple shape matching by incorporating accurate electronic properties, leading to more reliable predictions of binding strength and stability. The goal was not immediate identification of potent inhibitors, but a systematic assessment of how these rings interact with BChE. This foundational knowledge will inform the design and synthesis of larger, more complex molecules with enhanced binding affinity and selectivity, ultimately aiming to develop compounds to inhibit BChE activity and potentially slow Alzheimer’s progression.

## 1. Introduction

Drug discovery, at its core, is the identification of novel compounds exhibiting desirable biological activity. The concept of drug-like chemical space is ubiquitous in this endeavour, influencing every stage from initial screening library design to lead optimization. This space defines the chemical properties considered favorable for a molecule to become a viable drug candidate. Despite its central importance, a universally accepted and unambiguous definition of drug-like remains elusive. The term is often interpreted in various ways, ranging from exact substructural matches to known drugs to approximations based on similarity of full molecular structures or overall properties. Determining the thresholds for *close* or *similar* relies on a diverse array of computational techniques, spanning from simple one-dimensional (1D) properties like calculated log*P* (Clog*P*) and polar surface area (PSA) to more complex two-dimensional (2D) fingerprint similarity analyses and three-dimensional (3D) shape or electrostatic potential comparisons [1,2]. Furthermore, drug-likeness is not a binary attribute but rather exists on a continuum, representing a probability rather than a definitive true/false state [3].

**Scheme 1:**
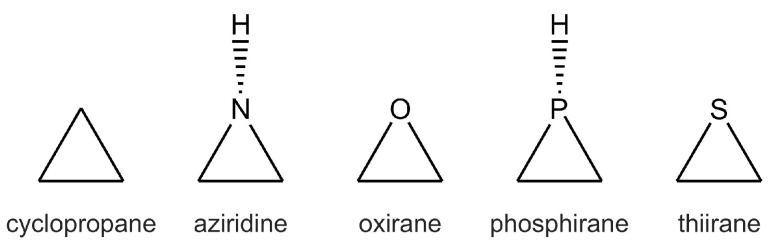
Chemical structures of three-membered cyclic compounds investigated as potential cholinesterase inhibitor scaffolds.

Early approaches to defining the drug-like space often focused on weighted combinations of whole-molecule properties. Simultaneously, 2D and 3D descriptors have been employed to identify compounds residing within this desirable chemical space [4]. Interestingly, analyses of molecules progressing through clinical trials reveal that compounds in later stages often exhibit significantly different property profiles compared to those ultimately approved as marketed drugs [5]. This suggests that the *ideal* drug-like space may be more nuanced and context-dependent than previously assumed. A crucial consideration in drug discovery is leveraging the knowledge embedded within existing drugs. Drug discovery efforts have historically relied on the iterative optimization of lead compounds, utilizing their molecular properties to guide structural modifications and generate novel pharmaceutical candidates [6]. However, initiating the discovery process with structurally novel scaffolds can be inefficient because there is limited pre-existing evidence of their therapeutic potential. Therefore, understanding the characteristics of successful drugs and defining optimal drug-like parameters are essential foundations for future research. Within a bioactive molecule, specific structural subunits often carry the essential features necessary for pharmacological activity [7]. These groups can function as pharmacophores or modulate overall bioactivity. Identifying and retaining these key scaffolds during chemical optimization and diversification is a successful strategy for maximizing the likelihood of clinical success. Three-membered rings (Scheme 1), cyclopropane, aziridine [8], oxirane [9,10], phosphirane [11], and thiirane [12] are such prevalent structural motifs found in molecules exhibiting diverse biological activities, including anticancer, antibacterial, and antiviral properties [13]. This literature data highlights the importance and significant potential of naturally occurring and synthetic compounds containing three-membered rings as valuable sources of drug leads with confirmed activities. Strained ring systems offer unique structural features and reactivity for the development of diverse pharmaceutical candidates. Furthermore, novel compounds incorporating these rings are being utilized and synthesized to create complex polyfunctional molecules with promising applications in drug discovery and design. However, a comprehensive review of the role of this complete class of three-membered ring structures in drug discovery is lacking.

Alzheimer’s disease (AD) still remains a significant therapeutic challenge. Since the acceptance of the cholinergic hypothesis, cholinesterases have been important targets for AD treatment, as cognitive impairment in AD is linked to deficient cholinergic transmission. Current treatments, while available (e.g., rivastigmine, donepezil, galantamine, memantine), often prove ineffective, prompting research into alternative strategies. Recent discoveries have revealed that cholinesterases can influence β-amyloid (Aβ) aggregation through a peripheral anionic site (PAS), renewing interest in these enzymes as therapeutic targets [14,15]. While acetylcholinesterase (AChE) has historically been the primary focus, butyrylcholinesterase (BChE) is gaining increasing attention due to its role in Aβ aggregation and its elevated concentration coinciding with decreasing AChE levels in later stages of the disease. Currently, five FDA-approved drugs inhibit AChE, but no BChE-selective drugs have been approved [16] although they may offer several advantages [17,18].

Traditional drug discovery approaches based on high-throughput screening (HTS) and synthesis have not consistently delivered significant efficiency gains. One potential reason is a focus on screening for drug-like compounds rather than lead-like compounds. Furthermore, lead optimization often increases molecular weight. A promising alternative strategy gaining momentum is fragment-based drug discovery (FBDD) [19,20]. This approach begins with very small molecules (fragments) that exhibit weak affinity for the target. Molecular size and weight are added only when they are accompanied by a corresponding increase in affinity, avoiding the creation of heavy, poorly active leads that are difficult to optimize. FBDD is particularly well-suited for targets where binding can occur at multiple sites within an enzyme, where joining two fragments binding to different sites can lead to synergistic increases in affinity.

Jencks explained that a molecule with two interacting parts contributes to free energy through both the interactions of those parts and the overall rotational and translational entropy of the molecule, where the affinity of the joined molecule should exceed the sum of the individual fragment affinities [21]. This is because the binding process involves a loss of rotational and translational entropy, and joining fragments reduces the entropic penalty. This paper, in part, explores the energetics of fragment linking, focusing on the loss of rigid-body entropy. We will analyze data from multiple-site binding simulations to estimate this energy cost and discuss the implications for FBDD, including the prevalence of multiple-site binding, the tractability of fragment optimization, the sensitivity of structure–activity relationships, and the difficulties encountered with HTS hits. Drug molecules need high affinity and selectivity for their targets, achieved through favorable binding enthalpy and entropy [22]. While optimizing entropy is relatively straightforward, maximizing enthalpy is difficult and time-consuming, often resulting in thermodynamically unbalanced molecules with suboptimal potency. New structure/activity relationship (SAR) approaches that explicitly consider both enthalpy and entropy are emerging to improve and accelerate the drug optimization process [23].

To accurately assess these energetic factors, we have employed the PM7 semi-empirical Hamiltonian to determine the corresponding heats of formation (Δ*H*_f_(g)) [24,25], with the method shown to outperform other semi-empirical approaches for organic molecules [26,27,28,29]. In direct comparison with its predecessor, PM6, and enhanced versions of the same Hamiltonian, the PM7 method performed slightly worse but offered robustness and versatility important for investigating interactions across a wide range of elements [26]. Moreover, the PM7 method was used to reproduce and even correct many structural features of various enzymes [30].

Furthermore, PM7 is readily deployable and applicable to a wide range of elements [31]. While PM7’s accuracy continues to improve, its ease of use and accessibility make it a valuable tool for high-throughput studies. We will validate PM7’s performance with additional data and provide insights into the energetics of fragment linking, ultimately shedding light on the challenges and opportunities in fragment-based drug discovery.

## 2. Materials and Methods

### 2.1. Active Site Model of BChE

Active site model of BChE was built using the following 127 aminoacids: Cys65, Cys66, Gln67, Asn68, Ile69, Asp70, Gln71, Ser72, Phe73, Pro74, Gly75, Phe76, His77, Gly78, Ser79, Glu80, Met81, Trp82, Asn83, Pro84, Asn85, Thr86, Asp87, Leu88, Trp112, Ile113, Tyr114, Gly115, Gly116, Gly117, Phe118, Gln119, Thr120, Gly121, Thr122, Ser123, Ser124, Leu125, His126, Val127, Tyr128, Asp129, Gly130, Asn145, Tyr146, Arg147, Val148, Gly149, Gly196, Glu197, Ser198, Ala199, Gly200, Ala201, Ala202, Ser203, Gln223, Ser224, Gly225, Ser226, Phe227, Asn228, Ala229, Pro230, Trp231, Ala232, Val233, Thr234, Arg242, Tyr282, Gly283, Thr284, Pro285, Leu286, Ser287, Val288, Asn289, Phe290, Gly291, Pro292, Asn322, Lys323, Asp324, Glu325, Gly326, Thr327, Ala328, Phe329, Leu330, Val331, Tyr332, Gly333, Ala334, Gly353, Leu354, Lys355, Ile356, Phe357, Phe358, Pro359, Gly360, Val393, Gly394, Tyr396, Asn397, Phe398, Pro401, Leu428, Pro429, Trp430, Pro431, Glu432, Trp433, Met434, Gly435, Val436, Met437, His438, Gly439, Tyr440, Glu441, Ile442, Glu443, Phe444, Val445, Phe446, Gly447. This active site model was used for quantum–chemical multiligand simultaneous docking, with single-point calculations on *Monte Carlo*-generated structures. For more accurate but also considerably more demanding quantum–chemical optimization of geometries and subsequent calculation of harmonic frequencies and thermodynamic quantities—standard enthalpies, entropies, and Gibbs free energies of formation—a smaller model of the active site was built and used: Asp70, Trp82, Asn83, Tyr114, Gly115, Gly116, Gly117, Gln119, Thr120, Gly121, Thr122, Leu125, Tyr128, Glu197, Ser198, Ala199, Ser224, Trp231, Pro285, Leu286, Ser287, Val288, Asn289, Asp324, Glu325, Gly326, Ala328, Phe329, Tyr332, Phe398, Met437, His438, Gly439, Tyr440, Ile442.

### 2.2. Quantum–Chemical Docking

To comprehensively explore the binding potential of the investigated small molecules within the BChE active sites, we employed a parallelized *Monte Carlo* quantum–chemical multiple-ligand docking protocol. This protocol, implemented in our in-house software, facilitates efficient sampling of the vast conformational space accessible to the ligands [32,33]. Given the structural rigidity of the investigated small molecules, which have no multiple accessible conformations at room temperature, structure generation focused exclusively on translational and rotational degrees of freedom within the enzymes’ active sites. It enabled prioritization by exploring binding poses without being constrained by the computational cost of flexible-ligand docking.

A total of 1,000,000 structures were generated during each simulation. Rigorous quality control measures were implemented, discarding all structures exhibiting atomic overlaps to ensure physically realistic binding poses. For all accepted structures, single-point enthalpy calculations were performed using the PM7 Hamiltonian [24]. This semi-empirical quantum–chemical approach provides a computationally efficient means of estimating binding energies and assessing the thermodynamic favourability of different binding poses at reasonable computational cost [28]. Binding energies within BChE active sites were then estimated. Following this initial energy assessment, full geometry optimization and harmonic frequency calculations were performed on the top 1000 structures ranked by their low energy values to refine the structures and obtain more accurate binding energies and other thermodynamic quantities.

To facilitate analysis and identify promising candidates, the resulting structures were subjected to hierarchical clustering based solely on their binding energies. This allowed us to identify clusters of similar binding poses and prioritize those with the lowest predicted energies. It was clear from the initial assessment that most of the 1000 structures are grouped around two main positions within the active site. The subsequent full geometry optimization yielded two local minima on the potential energy surface. Automated interaction analysis, coupled with visual inspection of the structures, helped identify key interactions between ligands and active-site residues, providing insights into the primary binding forces.

### 2.3. Geometry Optimizations and Calculation of Thermodynamic Quantities

Each of the top 1000 structures, ranked by their low energy values determined by quantum–chemical docking, was subjected to geometry optimization using the PM7 method in the Gaussian16 package [34]. Positions of the heavy atoms in the active site model were frozen to stabilize the model, whereas all hydrogen atoms were allowed to optimize. The small molecules were fully free during optimization to achieve the best possible placement within the active site. Following the geometry optimizations, harmonic frequency calculations were performed and analyzed. The reaction standard enthalpies, entropies, and Gibbs free energies of formation were calculated at *T* = 298.15 K and *p* = 101,325 Pa. From these values, corresponding standard enthalpies, entropy contributions at room temperature, and Gibbs free energies of binding [35] were estimated according to the following formulas for one docked molecule and two simultaneously docked molecules:

(1) $$\Delta_{b}G{}^{\circ}\left(\mathrm{three-membered}\;\mathrm{ring}\right)=\Delta_{f}G{}^{\circ}\left(\mathrm{active}\;\mathrm{site}+\mathrm{three-membered}\;\mathrm{ring}\right)\;\;\;\;\;-\Delta_{f}G{}^{\circ}\left(\mathrm{active}\;\mathrm{site}\right)-\Delta_{f}G{}^{\circ}\left(\mathrm{three-membered}\;\mathrm{ring}\right)$$

(2) $$\Delta_{b}G{}^{\circ}\left(2\;\mathrm{three-membered}\;\mathrm{rings}\right)=\Delta_{f}G{}^{\circ}\left(\mathrm{active}\;\mathrm{site}+2\;\mathrm{three-membered}\;\mathrm{rings}\right)\;\;\;\;\;-\Delta_{f}G{}^{\circ}\left(\mathrm{active}\;\mathrm{site}\right)-2\Delta_{f}G{}^{\circ}\left(\mathrm{three-membered}\;\mathrm{ring}\right)$$

## 3. Results

### 3.1. Quantum–Chemical Docking

The primary objective of these computational simulations was to identify compounds that exhibit both high binding affinity and selectivity for inhibition and BChE. Beyond simply identifying compounds possessing these desired pharmacological properties, elucidating their precise binding modes within the BChE active site is crucial. This structural information serves as a foundational framework for the rational design of larger, more complex BChE inhibitors. Specifically, understanding how these smaller compounds orient and interact within the active site provides critical insights for building and optimizing the 3D structures of potential drug candidates, facilitating targeted molecular design and improved inhibitory potency.

**Figure 1:**
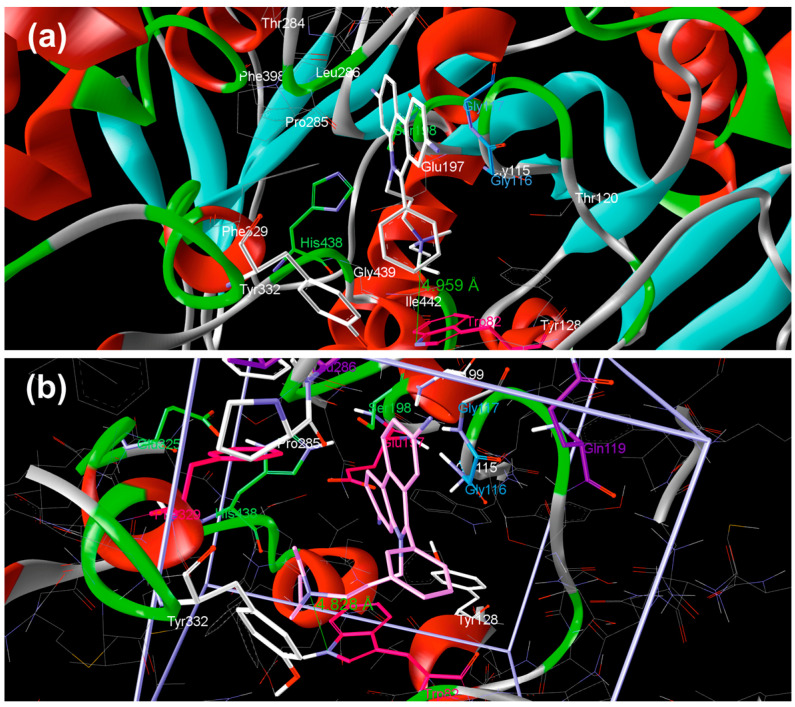
Placement of propidium fluorescent intercalating agent inside the active site of BChE: (**a**) crystal structure (white color) and (**b**) obtained by extensive quantum–chemical docking (violet color). Non-polar hydrogen atoms are omitted for clarity.

Such compounds represent promising candidates for the development of improved therapeutic strategies for Alzheimer’s disease, potentially offering a more targeted approach to modulating cholinergic neurotransmission and mitigating cognitive decline. This approach enables the identification and optimization of compounds exhibiting enhanced pharmacological properties, such as increased binding affinity and selectivity. For the initial and later stages of the process, quantum–chemical docking and geometry optimizations, along with the calculation of thermodynamic quantities, were performed using the PM7 method. The PM7 semi-empirical method was validated as a dependable tool for characterizing hydrogen bonds in complex organic molecules [36]. By successfully reproducing results from more computationally intensive ab initio methods such as Møller–Plesset or density functional theory, PM7 proves to be a reliable and efficient alternative for studying electron density trends at hydrogen-bond critical points. To further confirm the usability of the PM7 method for describing protein/small-molecule interactions, docking of a propidium fluorescent intercalating agent into the active site of BChE was performed and compared to the crystal structure [37]. The obtained results are presented in Figure 1.

Placement of the phenanthridine moiety is very similar, pointing to Ser198 and His438, whereas the distances between the charged nitrogen atom of propidium and the nitrogen atom of Trp82 were 4.959 and 4.828 Å for the crystal structure and the structure of minimal energy obtained by quantum–chemical docking.

#### 3.1.1. Docking of a Single Molecule

Molecular docking simulations of a single molecule for the tested compounds revealed consistent binding modes within the active site model. For the cyclopropane molecule, with no heteroatoms present in the ring structure, standard Gibbs free energies of binding for the two lowest energy clusters were calculated as −25.25 and −22.21 kJ mol^−1^ (Table 1). The standard Gibbs free energy of binding is the lowest for the phosphirane molecule (−143.90 kJ mol^−1^) due to the very strong interaction with Glu197, and then it rises in the order of aziridine (−98.50 kJ mol^−1^), oxirane (−50.69 kJ mol^−1^), and thiirane (−47.16 kJ mol^−1^). Binding energies in the second cluster are consistently higher, with the lowest value of standard Gibbs free energy for aziridine (−89.07 kJ mol^−1^), and then it increases for phosphirane (−64.05 kJ mol^−1^), oxirane (−38.99 kJ mol^−1^) and thiirane (−18.72 kJ mol^−1^). As expected, the entropy contribution at 298.15 K is similar for all compounds and stays in the window of −37.04 kJ mol^−1^ for cyclopropane to −50.94 kJ mol^−1^ for aziridine.

**Figure 2:**
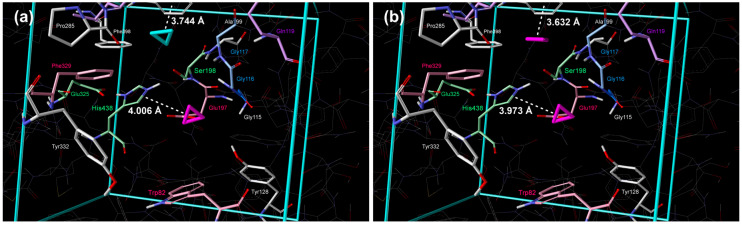
Placement of cyclopropane inside the active site of BChE obtained by extensive quantum–chemical docking: (**a**) single molecule representative of two lowest energy clusters (violet color: 1st cluster, turquoise: 2nd cluster) and (**b**) two molecules docked simultaneously, representative of the lowest energy cluster (violet color). Non-polar hydrogen atoms are omitted for clarity.

The positions of the molecules are shown in Figure 2a; the molecule in the 1st cluster, with the lowest standard Gibbs free energy of binding, was positioned in proximity to Ser198 and Glu197 (Figure 2a and Table 2). In the 2nd cluster, the molecule was placed in the acyl pocket near Trp231, Leu286, and Val288 (Figure 2a and Table 2).

**Table 2:** Distances between docked structures of three-membered rings in the active sites of BChE and selected amino acids (only 1 molecule was docked).

**Docked Molecule** **(1st Cluster)**	***r*** **/Å**				
	**Ser198**	**His438**	**Glu197**	**Trp82**	**Gly116**
cyclopropane	3.631 (C⋯O)	4.006 (C⋯N)	3.169 (C⋯O) 3.569 (C⋯O)	4.800 (C⋯C)	3.238 (C⋯N)
aziridine	**2.803 (N**⋯**O)**	4.027 (C⋯N)	3.123 (C⋯O) 3.758 (C⋯O)	5.863 (C⋯C)	**2.847 (N**⋯**N)**
oxirane	3.153 (O⋯O)	3.926 (C⋯N)	2.869 (C⋯O) 3.601 (C⋯O)	5.595 (C⋯C)	**2.748 (O**⋯**N)**
phosphirane	4.618 (P⋯O)	4.860 (P⋯O)	**1.778 (P**⋯**O)** 3.222 (P⋯O)	3.403 (C⋯C)	4.415 (P⋯N)
thiirane	4.262 (S⋯O)	3.574 (C⋯N)	3.093 (C⋯O) 3.119 (C⋯O)	3.945 (C⋯C)	3.561 (S⋯N)
**Docked Molecule** **(2nd Cluster)**	***r*** **/Å**				
	**Ser198**	**His438**	**Gly117**	**Ala199**	**Trp231**
cyclopropane	3.060 (C⋯O)	4.389 (C⋯N)	4.002 (C⋯N)	4.558 (C⋯N)	3.744 (C⋯C)
aziridine	**2.635 (N**⋯**O)**	4.550 (N⋯N)	**2.784 (N**⋯**N)**	**3.555 (N**⋯**N)**	3.460 (C⋯C)
oxirane	**2.769 (C**⋯**O)** **2.916 (C**⋯**O)**	4.352 (C⋯N)	**2.595 (O**⋯**N)**	**3.266 (O**⋯**N)**	3.240 (C⋯C)
phosphirane	3.249 (P⋯O)	4.898 (P⋯N)	3.956 (P⋯N)	4.229 (P⋯N)	3.439 (C⋯C)
thiirane	**2.609 (C**⋯**O)** **2.680 (C**⋯**O)**	3.623 (C⋯N)	4.245 (S⋯N)	5.536 (S⋯N)	3.477 (C⋯C)

Strong interactions are written in bold.

**Figure 3:**
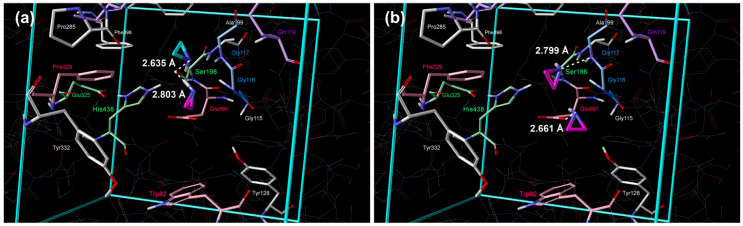
Placement of aziridine inside the active site of BChE obtained by extensive quantum–chemical docking: (**a**) single molecule representative of two lowest energy clusters (violet color: 1st cluster, turquoise: 2nd cluster) and (**b**) two molecules docked simultaneously, representative of the lowest energy cluster (violet color). Non-polar hydrogen atoms are omitted for clarity.

**Figure 4:**
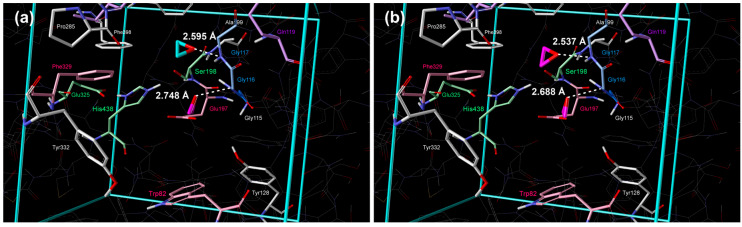
Placement of oxirane inside the active site of BChE obtained by extensive quantum–chemical docking: (**a**) single molecule representative of two lowest energy clusters (violet color: 1st cluster, turquoise: 2nd cluster) and (**b**) two molecules docked simultaneously, representative of the lowest energy cluster (violet color). Non-polar hydrogen atoms are omitted for clarity.

**Figure 5:**
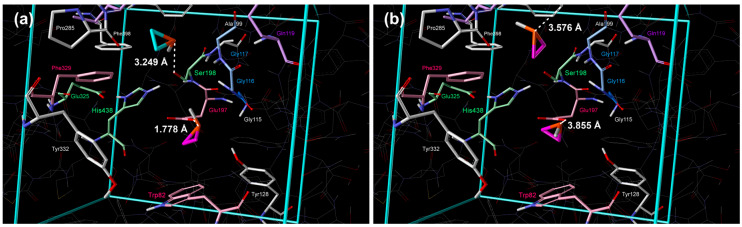
Placement of phosphirane inside the active site of BChE obtained by extensive quantum–chemical docking: (**a**) single molecule representative of two lowest energy clusters (violet color: 1st cluster, turquoise: 2nd cluster) and (**b**) two molecules docked simultaneously, representative of the lowest energy cluster (violet color). Non-polar hydrogen atoms are omitted for clarity.

**Figure 6:**
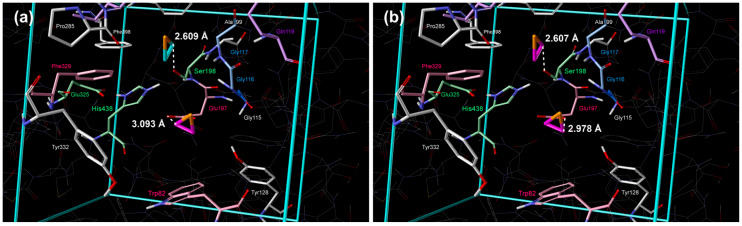
Placement of thiirane inside the active site of BChE obtained by extensive quantum–chemical docking: (**a**) single molecule representative of two lowest energy clusters (violet color: 1st cluster, turquoise: 2nd cluster) and (**b**) two molecules docked simultaneously, representative of the lowest energy cluster (violet color). Non-polar hydrogen atoms are omitted for clarity.

The position of molecules of strained heterocycles (aziridine, oxirane, phosphirane, and thiirane) in the 1st cluster is similar to the position of cyclopropane, but nitrogen, oxygen, and sulfur atoms are pointing towards Ser198, whereas the phosphorous atom of phosphirane was directed to Glu197 (Figure 3a, Figure 4a, Figure 5a and Figure 6a, Table 2). Additional hydrogen bonds form between the heteroatoms of aziridine and oxirane and the –NH group of Gly116. All molecules in the 2nd cluster have –CH_2_ interactions with the π-electrons of Trp231.

Position of molecules in the 2nd cluster is again similar to the position of cyclopropane in the 2nd cluster, but nitrogen, oxygen, and phosphorous atoms are pointing towards Ser198, whereas the sulfur atom of thiirane was directed away (Figure 3a, Figure 4a, Figure 5a and Figure 6a, Table 2). Additional hydrogen bonds form between the heteroatoms of aziridine and oxirane and the –NH group of Gly117 and the –NH group of Ala199.

#### 3.1.2. Simultaneous Docking of Two Molecules

Simultaneous docking of two molecules revealed significant insights into cooperative and competitive binding phenomena within the BChE active site (Table 3, Table 4, Figure 2b, Figure 3b, Figure 4b, Figure 5b, Figure 6b). Our results demonstrate that binding a second molecule can induce conformational changes, create new binding pockets, or cause steric clashes, thereby influencing overall binding affinity and stability. Notably, the strongest synergistic effect was observed with double occupancy by cyclopropane molecules, indicating a highly favorable cooperative interaction. Conversely, the binding of two phosphirane molecules exhibited the lowest level of cooperativity (Table 1 and Table 3).

This diminished cooperativity stems from a unique binding arrangement in which, upon dual occupancy, one phosphirane molecule remains near Glu197 while the second drifts, resulting in crowding of the oxyanion hole and subsequent steric interference (Figure 5b). Consistent with this observation, the enthalpic penalty for the second phosphirane ligand was the largest among the tested compounds. In contrast, aziridine displayed the smallest enthalpic loss for the second ligand, suggesting a more compatible geometry for dual binding and a greater potential for cooperative stabilization. These findings highlight the importance of considering multiligand binding scenarios to fully understand the complexities of inhibitor interactions with cholinesterases and to identify optimal combinations that maximize inhibitory potency.

## 4. Discussion

### 4.1. Quantum–Chemical Docking

This study utilized quantum–chemical docking to investigate the binding affinities and modes of a series of three-membered rings (cyclopropane, aziridine, oxirane, phosphirane, and thiirane) within the active site of human BChE. The results demonstrate a clear correlation between heteroatom composition and binding strength, providing valuable insights for the rational design of potent BChE inhibitors targeting AD. The consistent use of the PM7 semi-empirical method, previously validated against more computationally demanding ab initio approaches [28], offers an efficient and reliable pathway for exploring structure–activity relationships in this therapeutic context, not only for the investigated molecules but also for bigger and more complex molecular scaffolds.

#### 4.1.1. Docking of a Single Molecule

Quantification of binding affinities via calculated standard binding enthalpies and Gibbs energies revealed a trend: P ≪ N < O < S ≪ cyclopropane for the 1st lowest energy cluster and N ≪ P < O < cyclopropane < S for the 2nd energy cluster. This observed trend highlights the significant contribution of heteroatoms to BChE inhibition. The phosphirane molecule exhibited the strongest binding affinity, likely due to the strong interaction between its phosphorus atom and Glu197 in the 1st cluster (Table 1). After the quantum–chemical geometry optimization, the distance between the P atom of phosphirane and the O atom of Glu197 was 1.778 Å (Table 2 and Figure 5a), which is slightly larger than the length of the covalent P–O bond in phosphates [38]. The exceptionally low values of binding enthalpy and Gibbs free energy demonstrate a strong, enthalpy-driven interaction. This finding underscores the importance of designing ligands with functional groups that maximize strong interactions within the active site. While the cyclopropane molecule demonstrated the weakest binding affinity, serving as a useful baseline for comparison, the presence of heteroatoms consistently enhanced binding, supporting the hypothesis that these groups facilitate more favorable interactions with the enzyme.

Analysis of the docking poses reveals two dominant binding modes, consistent across all tested compounds. The primary cluster (1st cluster) positions the molecules in proximity to Ser198 and Glu197, suggesting this region is crucial for initial binding and stabilization. This spatial arrangement led to direct interactions with the nucleophilic Ser198 side chain and partial intrusion into the oxyanion hole, thereby disrupting the catalytic triad (Ser198–His438–Glu325). The consistent orientation of the heteroatoms towards Ser198 further reinforces the importance of this residue in mediating interactions. Interestingly, the 2nd cluster positions the molecules within the acyl pocket, where they interact with residues such as Trp231, Leu286, Val288, and Phe398. This suggests a degree of flexibility within the active site, allowing alternative binding orientations, potentially contributing to broader inhibitory activity. The observed –CH_2_ interactions with the π-electrons of Trp231 in the 2nd cluster pose indicate the importance of hydrophobic interactions in stabilizing the binding pose.

The formation of additional hydrogen bonds, particularly between the heteroatoms of aziridine and oxirane and Gly116/117, and Ala199, contributes to the enhanced stability of the binding poses within the 1st and 2nd clusters, respectively. This highlights the importance of incorporating groups that can participate in hydrogen-bonding networks to maximize binding affinity. These findings have important implications for the development of novel BChE inhibitors for the treatment of AD. By identifying specific residues involved in binding and understanding the preferred orientations of these compounds, we can guide the design of more complex molecules with improved binding affinity and selectivity. Whereas phosphirane demonstrated the most favorable raw binding energy, aziridine exhibited a compelling combination of good binding affinity and superior geometric alignment for effective catalysis disruption. These findings suggest that both phosphirane and aziridine represent promising scaffolds for the development of potent BChE inhibitors, albeit through distinct mechanisms.

#### 4.1.2. Simultaneous Docking of Two Molecules

Quantum–chemical multiligand simultaneous docking revealed some cooperative binding effects of two three-membered heterocyclic molecules within the BChE active site, providing insights into multiligand binding and its impact on inhibitory potential. Our results demonstrate that the binding of a second ligand is not necessarily additive; rather, it can probably induce changes in the active site and create new binding opportunities, or, conversely, lead to steric clashes, thereby fundamentally altering the overall binding affinity and stability.

Despite a favorable binding enthalpy (Δ_b_*H*° = −124.68 kJ mol^−1^, Table 3) for two cyclopropane molecules, which is comparable to that of two clusters of one molecule (Table 1), binding affinity is reduced due to a loss of configurational entropy. This entropy loss arises from the restricted motion of small cyclopropane molecules upon binding to the protein. Relatively modest negative entropy contribution (*T*_298.15 K_·Δ_b_*S*° = −80.15 kJ mol^−1^) is still bigger in absolute value than two clusters separately, resulting in an overall negative value of standard Gibbs energy of binding (Δ_b_*G*° = −44.54 kJ mol^−1^) with no synergistic effect. This suggests that the dual binding of cyclopropane is primarily driven by strong enthalpic interactions.

In contrast, the binding of two aziridine or two phosphirane molecules exhibited the lowest level of cooperativity. This diminished cooperativity is strongly linked to a unique binding arrangement, highlighted by the structural data (Figure 3 and Figure 5). While one aziridine or one phosphirane molecule remains proximate to Glu197, the other drifts closer toward the acyl pocket, thereby crowding the oxyanion hole and causing steric interference. It results in the largest enthalpic contribution (−244.39 kJ mol^−1^ and −233.51 kJ mol^−1^, respectively) amongst the tested compounds, directly contributing to the stronger overall binding that reflects in standard Gibbs energies of binding too (−146.07 kJ mol^−1^ and −140.66 kJ mol^−1^, respectively).

The most striking observation was the robust synergistic effect observed with thiirane molecules in double occupancy. Although the entropy contribution is the same as the sum of two individual clusters, the enthalpy contribution is significantly higher, resulting in the cooperative effect of approximately 30 kJ mol^−1^ (Table 3).

The variations in binding arrangements and resulting thermodynamic profiles observed across the different compounds underscore the importance of considering the specific chemical properties and geometries of potential ligands. Our data suggest that the ability of a second ligand to bind without disrupting existing favorable interactions, or to possibly induce a conformational change that creates new binding opportunities, is crucial for achieving synergistic inhibition. The distances observed in the tables (r/Å) confirm these interactions, specifically highlighting the altered interactions with residues such as Gly117, Ala199, and Trp231 for the second ligand. These findings have important implications for the rational design of multi-target inhibitors for cholinesterases. By carefully considering the potential for cooperative and competitive binding phenomena, it may be possible to develop ligand combinations that maximize inhibitory potency and overcome the limitations of single-ligand approaches.

Future research should prioritize a detailed investigation of binding modes and cooperative effects with larger, more complex ligands. Crucially, this requires a combined computational and experimental approach. Density functional theory (DFT) calculations will be essential for accurately predicting ligand binding poses and interactions within the active site of BChE. These in silico predictions should be validated and refined through molecular dynamics simulations to capture the dynamic interplay between multiple ligands and the protein [39]. Furthermore, in vitro studies assessing the impact of these compounds on BChE activity are vital for confirming computational predictions and evaluating their therapeutic potential.

## 5. Conclusions

This study employed a quantum–chemical multiple-ligand docking protocol to comprehensively investigate the binding potential of several strained compounds within the active sites of both AChE and BChE. Our results demonstrate that aziridine, oxirane, thiirane, and phosphirane all exhibit promising binding affinities and distinct interaction modes within the BChE active site, positioning them as potential scaffolds for the development of novel cholinesterase inhibitors. Single-molecule docking revealed consistent binding modes for aziridine, oxirane, and thiirane, disrupting the catalytic triad by interacting with Ser198 and partially intruding into the oxyanion hole. While phosphirane demonstrated the strongest raw binding energy, aziridine exhibited a superior geometric alignment for effective disruption of catalysis.

Crucially, the investigation of dual-ligand binding revealed significant insights into cooperative and competitive effects. Double occupancy by cyclopropane molecules displayed the strongest synergistic effect, indicating a highly favorable cooperative interaction. Conversely, phosphirane exhibited the lowest cooperativity, resulting from steric interference arising from a unique binding arrangement in which the second molecule drifted toward the oxyanion hole. Aziridine, in contrast, displayed the smallest enthalpic loss upon dual binding, suggesting a greater potential for cooperative stabilization.

These findings underscore the importance of considering multi-ligand binding in the design of cholinesterase inhibitors. The identification of aziridine and phosphirane as particularly promising scaffolds, coupled with the insights gained from dual-ligand docking, provides a strong foundation for the rational design of more complex inhibitors with enhanced potency and selectivity. Further research is warranted to optimize and combine the structures of these lead fragments and those of additional compounds, and to evaluate their in vitro and in vivo efficacy, with the ultimate goal of developing improved therapeutic strategies for Alzheimer’s disease and other cholinergic disorders.

## Figures and Tables

**Table 1:** Standard enthalpies, entropy contributions at room temperature, and Gibbs free energies of binding for docked structures of three-membered rings in the active sites of BChE (only 1 molecule was docked).

Single Docked Molecule	Δ_b_*H*°/kJ mol^−1^	*T*_298.15 K_·Δ_b_*S*°/kJ mol^−1^	Δ_b_*G*°/kJ mol^−1^
cyclopropane (1st cluster)	−65.56	−40.31	−25.25
cyclopropane (2nd cluster)	−59.25	−37.04	−22.21
aziridine (1st cluster)	−149.45	−50.94	−98.50
aziridine (2nd cluster)	−138.35	−49.28	−89.07
oxirane (1st cluster)	−99.11	−48.42	−50.69
oxirane (2nd cluster)	−84.60	−45.61	−38.99
phosphirane (1st cluster)	−194.03	−50.13	−143.90
phosphirane (2nd cluster)	−110.03	−45.98	−64.05
thiirane (1st cluster)	−90.81	−43.65	−47.16
thiirane (2nd cluster)	−60.21	−41.49	−18.72

**Table 3:** Standard enthalpies, entropies, and Gibbs free energies of binding for docked structures of three-membered rings in the active sites of BChE (2 molecules were docked simultaneously).

Two Docked Molecules	Δ_b_*H*°/kJ mol^−1^	*T*_298.15 K_·Δ_b_*S*°/kJ mol^−1^	Δ_b_*G*°/kJ mol^−1^
2×cyclopropane	−124.68	−80.15	−44.54
2×aziridine	−244.39	−98.32	−146.07
2×oxirane	−183.34	−95.75	−87.59
2×phosphirane	−233.51	−92.86	−140.66
2×thiirane	−186.45	−85.24	−101.21

**Table 4:** Distances between docked structures of three-membered rings in the active sites of BChE and selected amino acids (2 molecules were docked simultaneously).

**Docked Molecule** **(1st Molecule)**	***r*** **/Å**				
	**Ser198**	**His438**	**Glu197**	**Trp82**	**Gly116**
cyclopropane	3.587 (C⋯O)	3.973 (C⋯N)	3.185 (C⋯O) 3.546 (C⋯O)	4.893 (C⋯C)	3.227 (C⋯N)
aziridine	4.068 (N⋯O)	4.806 (N⋯N)	**2.661 (N**⋯**O)** 4.006 (N⋯O)	4.336 (C⋯C)	3.181 (N⋯N)
oxirane	3.288 (O⋯O)	4.236 (C⋯N)	2.745 (C⋯O) 3.541 (C⋯O)	5.267 (C⋯C)	**2.688 (O**⋯**N)**
phosphirane	4.605 (P⋯O)	4.558 (P⋯N)	3.855 (P⋯O) 4.456 (P⋯O)	3.544 (C⋯C)	3.965 (P⋯N)
thiirane	4.155 (S⋯O)	4.321 (C⋯N)	2.978 (C⋯O) 3.098 (C⋯O)	4.068 (C⋯C)	3.575 (S⋯N)
**Docked Molecule** **(2nd Molecule)**	***r*** **/Å**				
	**Ser198**	**His438**	**Gly117**	**Ala199**	**Trp231**
cyclopropane	3.204 (C⋯O)	4.733 (C⋯N)	3.660 (C⋯N)	4.113 (C⋯N)	3.632 (C⋯C)
aziridine	**2.664 (N**⋯**O)**	4.297 (N⋯N)	**2.799 (N**⋯**N)**	3.848 (N⋯N)	6.033 (C⋯C)
oxirane	**3.044 (C**⋯**O)** **2.870 (C**⋯**O)**	3.923 (C⋯N)	**2.537 (O**⋯**N)**	4.473 (O⋯N)	4.175 (C⋯C)
phosphirane	4.414 (P⋯O)	5.619 (P⋯N)	4.576 (P⋯N)	5.520 (P⋯N)	3.576 (C⋯C)
thiirane	**2.607 (C**⋯**O)** **2.782 (C**⋯**O)**	3.667 (C⋯N)	4.331 (S⋯N)	5.593 (S⋯N)	3.537 (C⋯C)

Strong interactions are written in bold.

## Data Availability

The raw data supporting the conclusions of this article will be made available by the authors on request.

## Abbreviations

The following abbreviations are used in this manuscript:

AChE	Acetylcholinesterase
BChE	Butyrylcholinesterase
AD	Alzheimer’s disease
Clog*P*	calculated log*P*, calculated octanol/water partition coefficient
PSA	polar surface area
PAS	peripheral anionic site
HTS	high-throughput screening
FBDD	fragment-based drug discovery
PM7	Parameterization Method 7, semiempirical quantum–chemical method
Δ_f_*G*°	standard Gibbs energy of formation
Δ_b_*G*°	standard Gibbs energy of binding
Δ_b_*H*°	standard enthalpy of binding
Δ_b_*S*°	standard entropy of binding
DFT	density functional theory
HPC	high-performance computing
